# Isoquinolinamine FX-9 Exhibits Anti-Mitotic Activity in Human and Canine Prostate Carcinoma Cell Lines

**DOI:** 10.3390/ijms20225567

**Published:** 2019-11-07

**Authors:** Jan Torben Schille, Ingo Nolte, Eva-Maria Packeiser, Laura Wiesner, Jens Ingo Hein, Franziska Weiner, Xiao-Feng Wu, Matthias Beller, Christian Junghanss, Hugo Murua Escobar

**Affiliations:** 1 Department of Medicine, Clinic III-Hematology, Oncology, Palliative Medicine, University of Rostock, 18057 Rostock, Germany; jan.torben.schille@tiho-hannover.de (J.T.S.); eva-maria.packeiser@tiho-hannover.de (E.-M.P.); laura.wiesner@tiho-hannover.de (L.W.); hein.jens@mh-hannover.de (J.I.H.); christian.junghanss@med.uni-rostock.de (C.J.); 2 Small Animal Clinic, University of Veterinary Medicine Hannover, 30559 Hannover, Germany; franziska.weiner@tiho-hannover.de; 3 Leibniz-Institute for Catalysis, University of Rostock, 18059 Rostock, Germany; xiao-feng.wu@catalysis.de (X.-F.W.); matthias.beller@catalysis.de (M.B.)

**Keywords:** isoquinolinamine, prostate cancer, cell line, human, dog, anti-mitotic, chemotherapy

## Abstract

Current therapies are insufficient for metastatic prostate cancer (PCa) in men and dogs. As human castrate-resistant PCa shares several characteristics with the canine disease, comparative evaluation of novel therapeutic agents is of considerable value for both species. Novel isoquinolinamine FX-9 exhibits antiproliferative activity in acute lymphoblastic leukemia cell lines but has not been tested yet on any solid neoplasia type. In this study, FX-9′s mediated effects were characterized on two human (PC-3, LNCaP) and two canine (CT1258, 0846) PCa cell lines, as well as benign solid tissue cells. FX-9 significantly inhibited cell viability and induced apoptosis with concentrations in the low micromolar range. Mediated effects were highly comparable between the PCa cell lines of both species, but less pronounced on non-malignant chondrocytes and fibroblasts. Interestingly, FX-9 exposure also leads to the formation and survival of enlarged multinucleated cells through mitotic slippage. Based on the results, FX-9 acts as an anti-mitotic agent with reduced cytotoxic activity in benign cells. The characterization of FX-9-induced effects on PCa cells provides a basis for in vivo studies with the potential of valuable transferable findings to the benefit of men and dogs.

## 1. Introduction

Prostate cancer (PCa) remains challenging in both humans and dogs. In men, PCa is the second most common malignancy diagnosed worldwide [1]. Localized PCa has a five-year survival rate of almost 100% [2], due to the availability of a broad range of curative treatment options including androgen deprivation therapy. However, 10% to 20% of PCa patients will progress to castrate-resistant PCa within five years of follow-up with over 84% of cases being metastatic [3]. Chemotherapeutics docetaxel and cabazitaxel as well as second-generation androgen receptor antagonists can prolong survival, but non-responding and developing resistances limit their efficacy [4,5,6,7]. Despite recent advances in treatment, the average survival time of metastatic castrate-resistant PCa remains at approximately three years. Accordingly, the development of novel therapeutic approaches addressing these challenges is essential [8].

Canine prostate adenocarcinomas share several characteristics with human metastatic castrate-resistant PCa such as age-dependent development, castration resistance, tumor progression, and metastatic pattern. Therefore, tumors seen in dogs are used as naturally occurring models of the human disease [9,10]. This is especially interesting as the canine tumors arises under presence of an immune system and both species are exposed to the same environmental risk factors [11,12]. PCa in dogs is a rare disease with an estimated prevalence below 1% based on necropsy studies. Canine patients are usually presented with late-stage highly malignant PCa without any curative treatment options [13,14,15,16]. Studies about chemotherapeutic treatment are rare, but chemotherapeutics in combination with non-steroidal anti-inflammatory drugs might improve survival [16]. Identification of novel agents against locally advanced, metastatic PCa can be beneficial for both species. Moreover, evaluation of these agents on canine tumors might accelerate the translation into human patients.

Amino-substituted isoquinoline structures are considered as tracers in positron emission tomography (PET) imaging [17,18,19] but also show a broader range of inhibitory [20,21,22] and cytotoxic effects e.g., activity against fungi [21], malaria [23], and cancer [22,24,25,26]. Recently, our group showed that novel synthesized isoquinolinamine FX-9 inhibits proliferation and induces apoptosis in human B- and T-acute lymphoblastic leukemia (ALL) cell lines, but displays no hemolysis on erythrocytes or cytotoxicity against non-neoplastic leukocytes. FX-9′s acting mechanism has not yet been revealed.

The aim of this study was to comparatively characterize the influence of FX-9 on two human and two canine prostate carcinoma cell lines. Human cell lines PC-3 and LNCaP are models for androgen receptor negative and androgen-sensitive tumors, respectively. Canine cell lines CT1258 and 0846 show different resistances towards common chemotherapeutic agents. This characterization is also the first of FX-9 on cells derived of solid tumors. Cellular effects of FX-9 on cell viability, total cell count and induction of apoptosis were determined. Besides morphological examination through May-Grünwald-Giemsa staining, live cell imaging was performed to record changes mediated by FX-9 over time. In addition, a first evaluation of FX-9 on non-malignant cells derived of solid tissues has been performed.

## 2. Results

### 2.1. Decrease of Cell Viability in Prostate Carcinoma Cells

**Figure 1:**
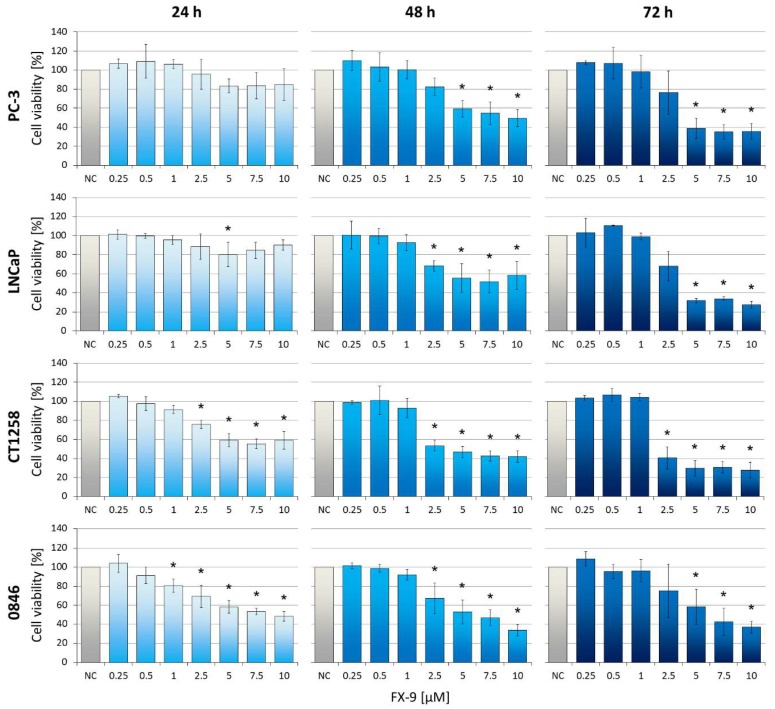
Human PC-3 and LNCaP and canine CT1258 and 0846 cells were exposed to increasing concentrations of FX-9 ranging from 0.25 µM to 10 µM. Cells were incubated for 24 h, 48 h, and 72 h. MTS assay was used to determine cell viability. The results are expressed as percentage of dimethyl sulfoxide (DMSO)-treated negative controls (NC, set to 100%). The diagrams show the mean ± standard deviation (SD) of three independent experiments. Significance of a treatment effect compared to the respective control was determined using Dunnett’s Multiple Comparison Test. *: *p* < 0.05.

**Figure 2:**
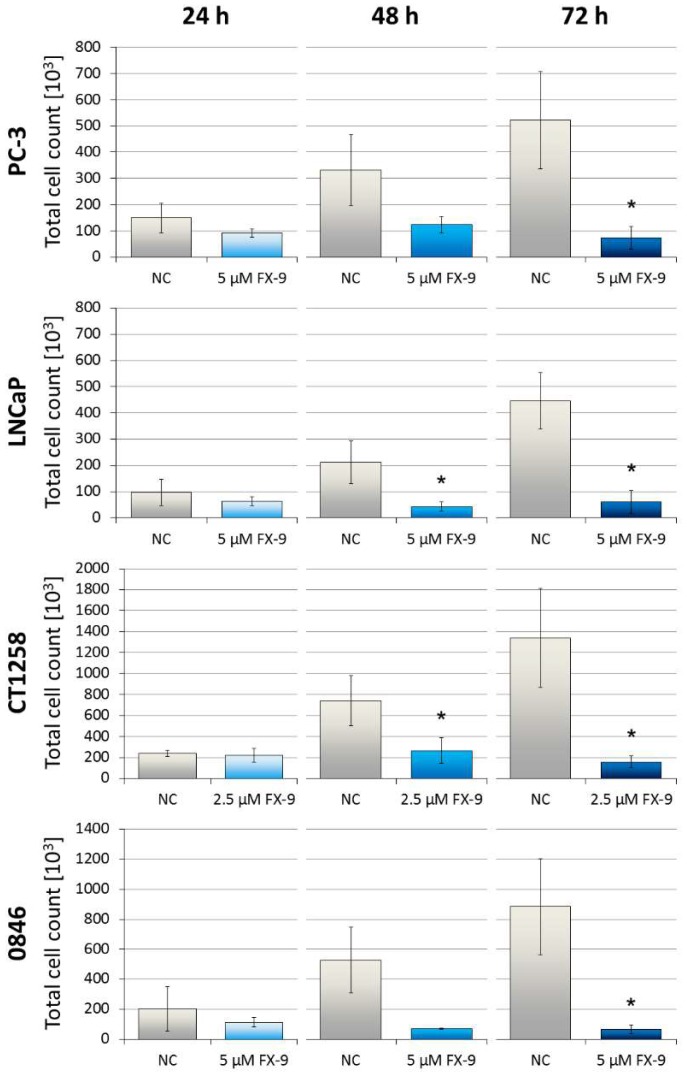
Prostate carcinoma cells lines were exposed to either 5 µM FX-9 (PC-3, LNCaP and 0846) or 2.5 µM FX-9 (CT1258) based on MTS assay for 24, 48, and 72 h. The results are expressed as total counted cells in the thousands via an automatic cell counter. The diagrams show the mean ± SD of three independent experiments. Significance of a treatment effect compared to the respective DMSO-treated negative control (NC) was determined using Student’s *t*-test. *: *p* < 0.05.

Dose- and time-dependent reduction of cell viability can be observed in all PCa cell lines post FX-9 incubation (Figure 1). Dosages of 5 µM and upwards caused significant inhibition in all cell lines after 48 h and 72 h compared to the negative controls. CT1258 was the only cell line with a significant inhibition starting at 2.5 µM of FX-9 72 h post application. At 10 µM FX-9, cell viability was determined between 27.4% (LNCaP) and 37% (0846). To further quantify viability of cells exposed to FX-9, the total number of cells were counted post FX-9 incubation compared to controls. A significant decrease in cell counts was observed after FX-9 exposure in all PCa cell lines after 72 h (Figure 2). Differences to controls were even more pronounced than seen in the MTS assay. While the control cells showed a steady increase in cell number over time, FX-9 incubated cells stagnated or decreased in their total cell counts ranging from 7.6 to 14.1% of control values after 72 h.

### 2.2. Morphological Changes in Prostate Carcinoma Cells

**Figure 3:**
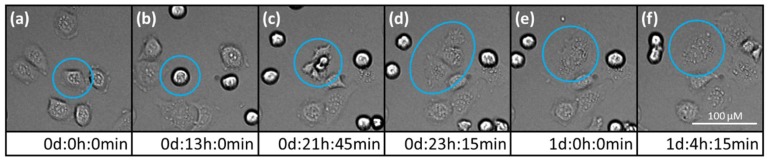
PC-3 cell undergoing mitotic slippage during 10 µM FX-9 exposure. Pictures show the same image section and cell (blue circle). (**a**) start of live cell imaging; diploid cell; (**b**) cell becomes round/detached for proliferation; (**c**) cell reattaches to surface at the end of cell cycle; (**d**) almost complete cytokinesis of daughter cells; (**e**) cytokinesis failed; daughter cells merge again; (**f**) survival of a tetraploid cell. Please check Supplementary Materials for the full movie.

Live cell imaging displayed an inhibited cell proliferation after 5 and 10 µM FX-9 exposure (and additionally after 2.5 µM for CT1258). Compared to the controls, the total number of cells was drastically reduced after 72 h. During incubation time, two distinct cell fates were observed. Induction of cell death occurred within the four PCa cell lines seen by the formation of apoptotic bodies. This induction of apoptosis took place during cell proliferation (round/detached cells). Secondly, at the end of the cell cycle, cytokinesis appears to fail in some cells leading to the formation of enlarged polyploid cells (Figure 3). Both effects occurred more often with higher FX-9 concentrations. Movies of the controls and of the four carcinoma cell lines incubated with FX-9 are given as Supplementary Materials (Movies S1–S13).

**Figure 4:**
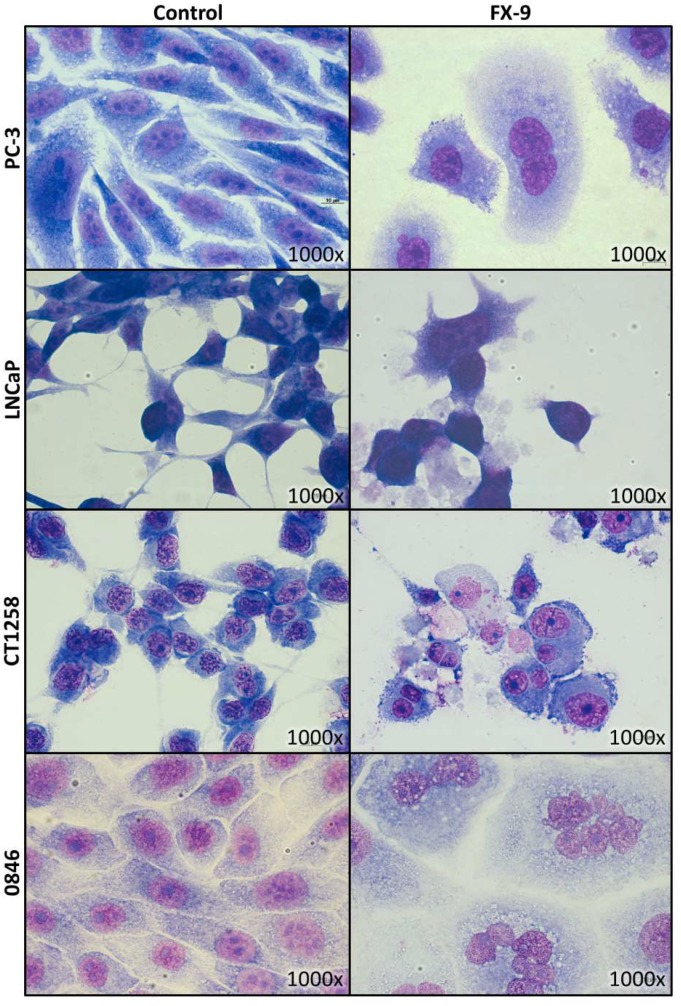
Human (PC-3, LNCaP) and canine (CT1258, 0846) cells were grown on microscope slides and incubated with 5 µM FX-9 for 72 h (2.5 µM in case of CT1258). Slides were stained via May-Grünwald-Giemsa staining. Representative pictures are displayed.

For May-Grünwald-Giemsa staining, the carcinoma cell lines were exposed to either 5 µM FX-9 or 2.5 µM in case of CT1258 based on MTS assay results. The staining revealed an altered cytomorphology in the tested cell lines (Figure 4). After exposure to FX-9, remaining cells tended to aggregate and lost their distinct shapes becoming round to pleomorphic. Enlarged cells with multiple nuclei could be observed, confirming live cell imaging observations of formation of polyploid cells through cell cycle disturbance.

### 2.3. Induction of Apoptosis in Prostate Carcinoma Cells

**Figure 5:**
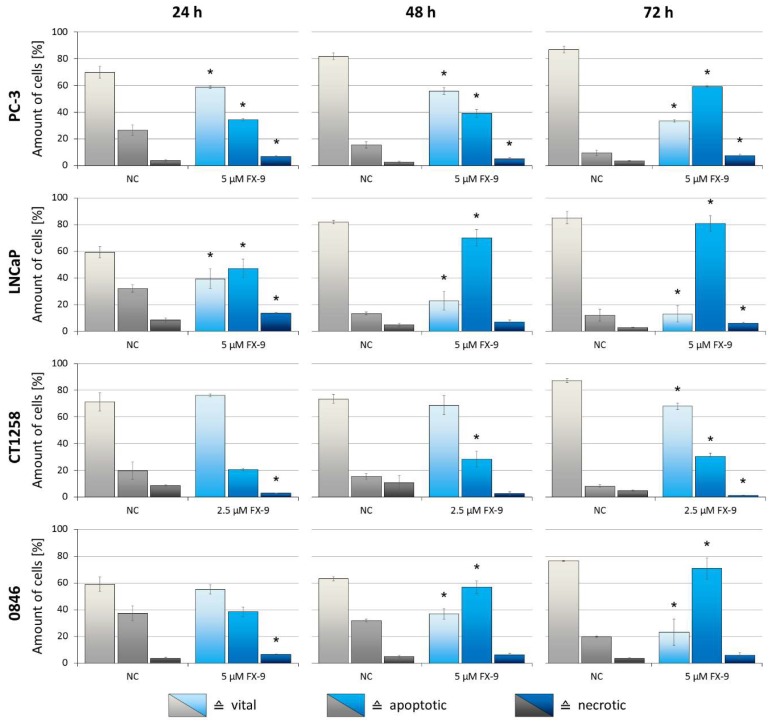
Prostate carcinoma cells lines were exposed to either 5 µM FX-9 (PC-3, LNCaP and 0846) or 2.5 µM FX-9 (CT1258) based on MTS assay for 24, 48 and 72 h. Analysis of apoptosis was performed using Annexin V-FITC (AV) and TO-PRO-3 Iodide (TP3) staining with subsequent flow cytometry analysis. Rates of vital (AV−, TP3−), apoptotic (AV+, TP3−) and necrotic cells (AV+/−, TP3+) are displayed as percentage of total amount of cells. The diagrams show the mean ± SD of three independent experiments. Significance of a treatment effect compared to the respective DMSO-treated negative control value (NC) was determined using the Student’s *t*-test. *: *p* < 0.05.

Consistently with live cell imaging observations, FX-9 exposure caused significant induction of apoptosis in all carcinoma cell lines (Figure 5). Within the negative controls, the amount of vital cells increased over time. On the contrary, the amount of apoptotic cells increased after FX-9 incubation, while the amount of necrotic cells remained relatively stable. For the three cell lines exposed to 5 µM FX-9, the amount of non-vital cells reached 66.7% (PC-3), 87% (LNCaP) and 76.8% (0846) after 72 h. Induction of apoptosis was less pronounced in CT1258 (exposed to 2.5 µM), but still significant.

### 2.4. Cell Cycle Arrest

After 12 h exposure to 5 µM FX-9, a fraction of cells showed irregular amount of DNA content (less than cells in G0/G1 phase or more than cells in G2/M phase). The effect was more pronounced in the faster proliferating 0846 cell line. These cells were excluded from the evaluation and remaining population was set to 100%. 

The amount of cells in the G0/G1 phase and in the S phase significantly decreased post FX-9 incubation (Table 1) in both cell lines. Accordingly, the amount of cells in the G2/M phase prior to cell division significantly increased from 39.4%/38.8% to 49.0%/49.8%.

### 2.5. Effects on Benign Cells

**Figure 6:**
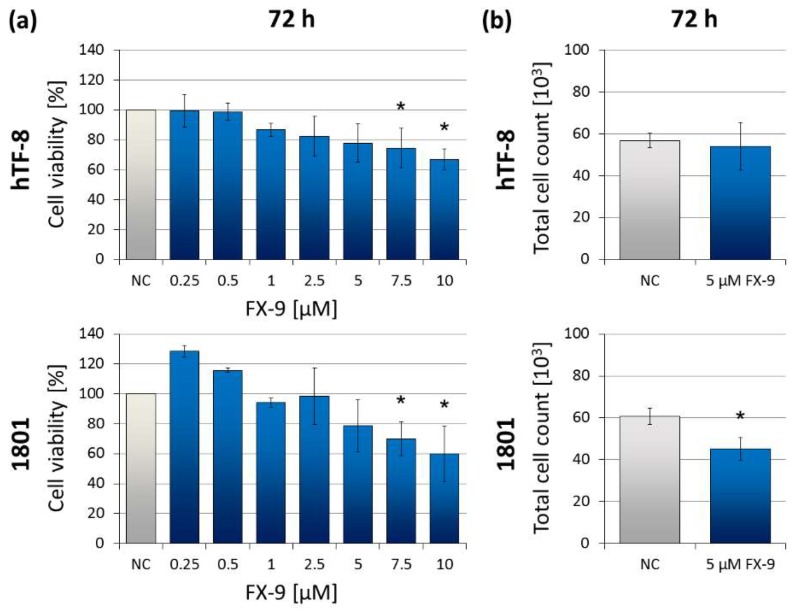
(**a**) human fibroblasts (hTF-8) and canine chondrocytes (1801) were exposed to increasing concentrations of FX-9 ranging from 0.25 µM to 10 µM for 72 h. MTS assay was used to determine cell viability. The results are expressed as percentage of DMSO-treated negative controls (NC, set to 100%). The diagrams show the mean ± SD of three measurements. Significance of a treatment effect compared to the control was determined using Dunnett’s Multiple Comparison Test. *: *p* < 0.05. (**b**) hTF-8 and 1801 were exposed to 5 µM FX-9 for 72 h. The results are expressed as total counted cells in thousands via automatic cell counter. The diagrams show the mean ± SD of three independent experiments. Significance of a treatment effect compared to the respective DMSO-treated negative control (NC) was determined using Student’s *t*-test. *: *p* < 0.05.

To further characterize the effect of FX-9 on non-malignant cells, human fibroblasts (hTF-8) and canine chondrocytes (1801) were exposed to FX-9. Concentrations up to 5 µM (effective concentration in the carcinoma cell lines) did not significantly reduce cell viability after 72 h of incubation (Figure 6). Cell viability was significantly reduced starting with concentrations of 7.5 µM in both cell types. Viability was reduced to 66.9% (hTF-8) and 60.1% (1801) with 10 µM FX-9. Total cell count of hTF-8 cells showed no significant change after FX-9 exposure; 1801 count decreased to 74.4% of non-treated control.

**Figure 7:**
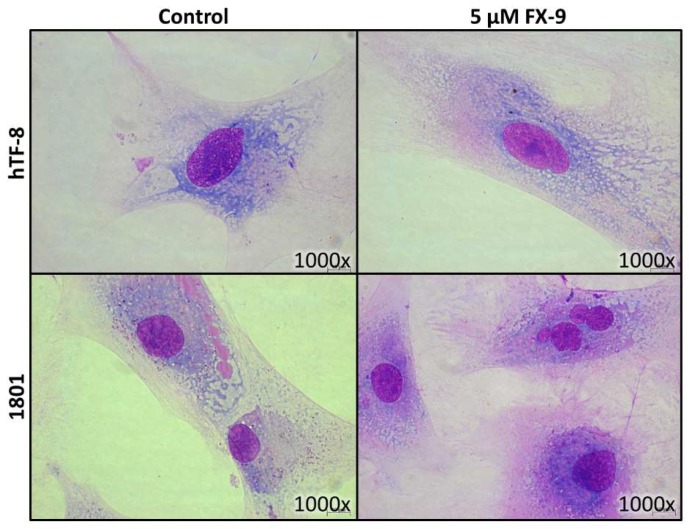
hTF-8 and 1801 cells were grown on microscope slides and incubated with 5 µM FX-9 for 72 h. Slides were stained via May-Grünwald-Giemsa staining. Representative pictures are displayed.

**Figure 8:**
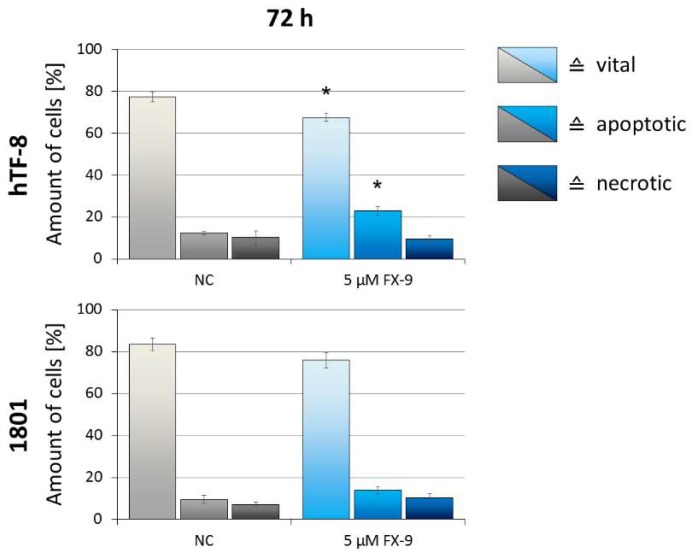
hTF-8 and 1801 cells were exposed to 5 µM FX-9 for 72 h. Analysis of apoptosis was performed using Annexin V-FITC (AV) and TO-PRO-3 Iodide (TP3) staining with subsequent flow cytometry analysis. Rates of vital (AV−, TP3−), apoptotic (AV+, TP3−), and necrotic cells (AV+/−, TP3+) are displayed as a percentage of total amount of cells. The diagrams show the mean ± SD of three independent experiments. Significance of a treatment effect compared to the respective DMSO-treated negative control value (NC) was determined using Student’s *t*-test. *: *p* < 0.05.

Cell morphology did not change through FX-9 exposure (Figure 7). Both cell types kept their shape and an intact cytoplasmic border. The only observable difference post FX-9 incubation was the formation of a small number of multinucleated cells. The amount of vital cells decreased from 77.3% to 67.5% in hTF-8 after 72 h of incubation with FX-9 (Figure 8). Accordingly, the amount of apoptotic cells increased from 12.4% to 23%. The percentage of necrotic cells did not change. Chondrocytes 1801 showed no significant change in vital/apoptotic/necrotic ratios 72 h post FX-9 exposure.

Compared to the benign cells, the PCa cells displayed significant different values for almost all cell lines and assays with concentrations of 5 µM FX-9 and upwards (Table 2).

## 3. Discussion

Within this study, the effects of isoquinolinamine FX-9 were comparatively evaluated on two human and two canine prostate carcinoma cell lines and additional non-malignant cells. The human PC-3 and LNCaP cell lines are well described and commonly used PCa cell lines, both established from metastases of adenocarcinomas. PC-3 is androgen receptor negative, representing a model for castrate-resistant prostate cancer. Contrary, LNCaP is androgen-sensitive [27,28]. Comparable canine PCa cell lines are rare. Herein, CT1258 and 0846 were used, two canine cell lines established from metastatic adenocarcinomas by our group. CT1258 has been characterized previously [29,30,31,32]. Complete characterization of cell line 0846 has not been published yet, but 0846 was already used in some studies [33,34]. CT1258 is more resistant against common chemotherapeutic agents like doxorubicin and carboplatin, while 0846 is more sensitive.

FX-9 significantly reduced cell viability and total cell count, and induced apoptosis in all four prostate carcinoma cell lines in a dose- and time-dependent manner. Reduction in cell viability was less pronounced in the PCa cell lines compared to ALL cell lines previously reported [35]. Exposed to 10 µM of FX-9, there was still around 30% of cell viability left compared to the controls. In comparison, ALL cell’s viability was reduced to 0.6% and 13.6% at the same concentration [35].

Induction of cell death in the PCa cell lines was comparable to ALL cells with rates of non-vital cells ranging from 66.7% to 87% after 72 h (5 µM FX-9 exposure). Interestingly, the amount of necrotic cells stayed low during FX-9 incubation (comparable to non-incubated controls), while only the amount of apoptotic cells drastically increased. Induction of apoptosis in anti-cancer approaches has significant advantages over necrotic induction, as apoptotic cells do not release damage-associated molecular pattern molecules and, therefore, do not induce inflammation and tissue damage [36,37]. However, massive apoptosis through cytotoxic therapies can overexert the in vivo clearance mechanisms for apoptotic cells, which then become secondary necrotic [36].

Interestingly, despite the low induction of apoptosis in CT1258 (exposed to 2.5 µM FX-9), total cell count is as much affected as in the other malignant cells (exposed to 5 µM FX-9) with much stronger induction of apoptosis. It appears that a concentration of 2.5 µM FX-9 is sufficient in inhibiting or at least delaying proliferation, but simultaneously induces only low apoptosis. This might also explain why CT1258 is the only cell line with significant decrease in cell viability at 2.5 µM FX-9. CT1258 is the fastest proliferating cell line of the four PCa cell lines (displayed in the highest absolute cell count after 72 h despite the same starting counts). The fast proliferating DMSO-treated CT1258 control cells increase absolute cell viability more than in the other cell lines, leading to the lowest relative cell viability of FX-9 exposed cells in comparison.

Live cell imaging confirmed strong induction of cell death and generation of apoptotic bodies during FX-9 incubation, consistent with apoptosis measurements by flow cytometry. In addition, giant cells formed after proliferation. May-Grünwald-Giemsa staining revealed these giant cells as intact and enlarged multinucleated cells. These cells have not been described in the ALL cell lines after FX-9 exposure [35] and might explain the higher cell viability in PCa compared to ALL cell lines post FX-9 incubation despite similar induction of cell death in both neoplasia. 

The benign fibroblasts and chondrocytes were less sensitive towards FX-9. Contrary to the carcinoma cell lines, exposure to 5 µM FX-9 did not significantly reduce cell viability. In addition, total cell count and induction of apoptosis were significantly less affected compared to the PCa cell lines. Benign cell morphology was also less altered. However, similar to the PCa cell lines, some multinucleated cells formed.

These polyploid cells usually form through cell cycle stress following exposure to cancer therapeutic agents like DNA or spindle damaging substances [38,39]. Cell cycle analysis showed cells with irregular DNA content already after 12 h of FX-9 incubation, especially within the faster proliferating 0846 cell line. Nonetheless, the cell cycle analysis also revealed an enrichment of cells in G2/M cell cycle phase, suggesting a cell cycle arrest in this phase.

Given the reduced effect of FX-9 on slower proliferating benign cells, as well as the clear impact on cell cycle (induction of apoptosis during proliferation, G2/M phase enrichment and formation of multinucleated cells), FX-9 induces its anti-cancer effects in an anti-mitotic manner. Induced G2/M-phase cell cycle arrest has been shown for some isoquinolinamine agents already [24,25,26] partly due to tubulin polymerization inhibition. Prolonged mitotic arrest will eventually lead to cell death by apoptosis as detected for the PCa cell lines exposed to FX-9. 

However, cells might overcome mitotic arrest, a process referred to as mitotic slippage [39,40]. These cells fail cytokinesis and remain in a tetraploid state, matching the enlarged multinucleated cells observed by May-Grünwald-Giemsa staining. They usually are senescent but bear a tumorigenic potential as their proliferation can lead to aneuploid cells, a hallmark of cancer [38,40,41,42]. Mitotic slippage might be a limiting factor and its potential risks need to be further evaluated.

In conclusion, FX-9 significantly induces apoptosis in human and canine PCa cell lines. The mediated effects and the efficacy of FX-9 were highly comparable between the used PCa cell lines, regardless of their origin. As expected for an anti-mitotic agent, androgen receptor negative cell line PC-3 as well as androgen-sensitive cell line LNCaP were affected. Therefore, the agent might be used in androgen-sensitive as well as castrate-resistant PCa subtypes. As its cytotoxicity was drastically reduced against benign cells, FX-9 possibly causes only low adverse effects in vivo. FX-9 appears to be a worthwhile agent for further evaluation as potential treatment of human and canine PCa. 

The characterization of FX-9-induced effects on PCa cells provides a basis for further in vivo studies. In a next step, a first experimental pharmacokinetic study needs to be performed to characterize the mediated effects and potential adverse reactions. If successful, an application in dogs with spontaneously occurring PCa could allow for evaluating the compound in the presence of an immune system. Consequently, therapy with FX-9 in dogs can provide valuable information for the animal itself, but also accelerate the translation into humans.

## 4. Materials and Methods 

### 4.1. Isoquinolinamine FX-9

**Figure 9:**
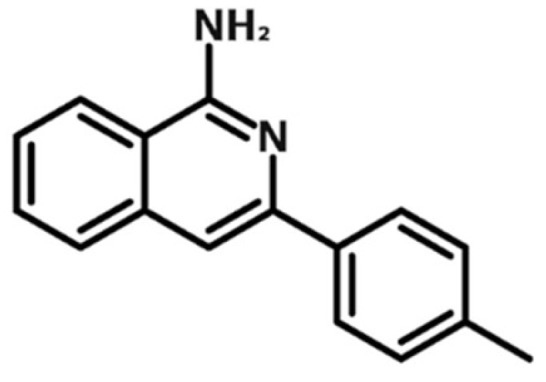
Chemical structure of isoquinolinamine FX-9.

Synthesis of isoquinolines has been previously described by us [43]. The chemical structure of FX-9 is displayed in Figure 9. FX-9 used in this study was synthesized during the original synthetic methodologies studies. The substance was dissolved in dimethyl sulfoxide (DMSO, Merck KGaA, Darmstadt, Germany) and the stock solutions (10 mM) were stored at −20 °C. For experimental use, the agent was freshly prepared from stock solution.

Different FX-9 concentrations and incubation times were used and compared against DMSO-treated controls as FX-9 itself was dissolved in this solvent. The used DMSO concentrations of 0.1% (*v*/*v*) were equivalent to the highest DMSO doses in the FX-9-treated samples to ensure that no possible effects of the solvent are measured.

### 4.2. Cell Lines and Cultivation

PC-3 [44], an androgen-independent cell line [45], and LNCaP [46], an androgen-sensitive cell line [45], were used for the evaluation on human prostate carcinoma cells. Both cell lines were obtained from DSMZ (German Collection of Microorganisms and Cell Cultures GmbH). As canine cell lines, two lines established by us from prostatic adenocarcinomas were used. Cell line TihoDProAdcarc1258 (CT1258) [29] was generated from a 9.7-year-old intact male Briard. TihoDProAdcarc0846 (0846) was generated from a 6.3-year old intact German Rough Haired Pointer. The benign human primary cell culture hTF-8 was obtained from a Tenon’s capsule during routine glaucoma surgery from a donor, who had given prior consent, following the declaration of Helsinki [47]. The primary cell culture of canine chondrocytes TiHoDKneeChon1801 (1801) was established from a 14-year-old, female, castrated mixed-breed. Human hTF-8 cells were used in passage eight and 1801 cells were used in passage ten. Early passages were used to mimic in vivo situation.

Human and canine carcinoma cell lines were cultivated in medium 199 (Live Technologies GmbH, Darmstadt, Germany), hTF-8 was cultivated in Dulbecco’s MEM/Ham’s F-12 (1:1) (Biochrom, Berlin, Germany) and 1801 in DMEM/Ham’s F-12 (1:1) with additional 1 mM l-glutamine and 10 μg/mL l-ascorbic acid (Biochrom). All media were supplemented with 10% of FBS Superior (Biochrom) and 2% penicillin/streptomycin (Biochrom). Cells were cultivated at 37 °C in a humidified atmosphere of 5% CO_2_. All cells tested negative for mycoplasma.

### 4.3. MTS Assay

Cell viability in response to FX-9 incubation was evaluated using CellTiter 96^®^ AQueous One Solution Cell Proliferation Assay (Promega, Fitchburg, MA, USA). The assay is a colorimetric method for determining the number of viable cells. The solution reagent contains the tetrazolium compound MTS (3-(4,5-dimethylthiazol-2-yl)-5-(3-carboxymethoxyphenyl)-2-(4-sulfophenyl)-2H-tetrazolium) that is bioreduced by cells into a colored formazan product. The quantity of formazan product is directly proportional to the number of living cells in culture. 

The four carcinoma cell lines as well as benign hTF-8 and 1801 cells were seeded in 96-well plates (7500 cells per well) and incubated overnight in 150 μL culture medium. On the next day, the cells were exposed to different concentrations of FX-9 (0.25 µM, 0.5 µM, 1.0 µM, 2.5 µM, 5.0 µM and 10 µM). Control cells were exposed to 0.1% DMSO. The MTS assay was carried out after 24 h, 48 h, and 72 h for the PCa cell lines and after 72 h for the benign cells according to the manufacturer’s protocol. The reaction products were quantified by measuring the absorbance at 490 nm using a Multi-Mode Reader Synergy 2 (BioTek Instruments, Winooski, VT, USA). For each FX-9 concentration and control, the mean value of four measured wells was used per experiment. Each experiment was carried out in biological triplicates.

### 4.4. Cell Count Analysis

The four carcinoma cell lines were seeded in 6-well plates (120 × 10^3^ cells per well) in 3 mL culture medium and incubated overnight. On the next day, FX-9 incubation was started (PC-3, LNCaP and 0846 with 5.0 µM FX-9 and CT1258 with 2.5 µM). After 24 h, 48 h, and 72 h the cells were harvested and counted via automatic cell counter (CellometerTM Auto T4, Nexcelom Bioscience, Lawrence, MA, USA). For the benign cells, hTF-8 and 1801, 60 × 10^3^ cells were seeded per well and treated with 5 µM FX-9 on the next day. Cells were counted after 72 h. For each FX-9 concentration and the controls, three wells were measured, and the mean value was used per experiment. Each independent experiment was performed three times.

### 4.5. Live Cell Imaging

PC-3, LNCaP, CT1258, and 0846 cells were seeded in 96-well plates (10 × 10^3^ cells per well) and incubated overnight in 150 μL of culture medium. On the next day, the culture medium was replaced by new mediums including 5 µM or 10 µM FX-9 (additionally 2.5 µM for CT1258) or 0.1% DMSO as control. The cells were incubated for 72 h in 150 μL in a live cell imaging system (DMI 6000 B, Leica Microsystems, Wetzlar, Germany) at 37 °C with 5% CO_2_ humidified atmosphere. A picture of each well was captured every 15 min during the incubation time and single pictures were combined to live cell imaging movies.

### 4.6. May-Grünwald-Giemsa Staining

Morphological changes mediated by FX-9 exposure were analyzed through May-Grünwald-Giemsa staining. The four PCa cell lines as well as benign hTF-8 and 1801 cells were seeded in 60 cm^2^ cell culture dishes (approx. 1 × 10^6^ cells per dish), which contained two microscope slides. After the cells reached about 80% confluence, the microscope slides were transferred to new dishes with FX-9 or DMSO for the control cells. Based on MTS assay PC-3, LNCaP and 0846 were incubated with 5.0 µM FX-9 and CT1258 with 2.5 µM FX-9 for 72 h. Benign cell lines hTF-8 and 1801 were also exposed to 5 µM FX-9 for 72 h. Microscope slides were air dried at room temperature. The slides were stained in May-Grünwald’s eosine-methylene blue solution (Merck, Darmstadt, Germany) for 200 s and rinsed with double distilled water. Afterwards, they were stained for 10 min in Giemsa’s azur eosin methylene blue solution (Merck, Darmstadt, Germany) and rinsed thoroughly with double distilled water again twice. The slides were left to air dry before analysis.

### 4.7. Analysis of Apoptosis

Determination of apoptosis rates was carried out by flow cytometry using a MACSQuant^®^ Analyzer 10 (Miltenyl Biotec, Bergisch Gladbach, Germany). Cells were stained with the fluorescence dyes Annexin V-FITC and TO-PRO-3. Annexin V binds to the membrane phospholipid phosphatidylserine, when it is translocated to the cell surface during apoptosis. TO-PRO-3 is unable to enter vital and apoptotic cells but stains necrotic cells by binding to cellular nucleic acids. 

The four PCa cell lines were seeded in 6-well plates (120 × 10^3^ cells per well) and incubated in 3 mL culture medium overnight. The culture medium was replaced by new medium including FX-9 (5.0 µM for PC-3, LNCaP and 0846 and 2.5 µM for CT1258) or DMSO as control. After 24 h, 48 h, and 72 h, the medium was collected saving the non-adherent cells fraction to count all treated cells regardless their condition. Remaining adherent cells were also harvested and pooled with the medium cells fraction. Cells were pelleted and resuspended in 500 µL binding buffer (Annexin V-FITC Detection Kit plus, PromoCell, Heidelberg, Germany). In the next step, 250 μL of the cell suspension was filtered with a 70 µm filter and 2.5 μL of Annexin V-FITC (PromoCell) and 0.5 μL of TO-PRO-3 Iodide (Thermo Fisher Scientific, Waltham, MA, USA) were added per sample and vortexed briefly. Before measurement, the samples were incubated at room temperature for 5 min. For the benign cells hTF-8 and 1801, 60 × 10^3^ cells were seeded per well and treated with 5 µM FX-9 on the next day. Flow cytometry analysis was performed after 72 h. The mean value of three measured wells was used per experiment. Each experiment was carried out in biological triplicates. Results were analyzed with FlowJo Version 7.6.5 (FlowJo, Ashland, OR, USA).

### 4.8. Cell Cycle Analysis

Changes within the rates of the different cell cycle phases (G0/G1, S and G2/M) after FX-9 incubation were determined by flow cytometry using a MACSQuant^®^ Analyzer 10 (Miltenyl Biotec, Bergisch Gladbach, Germany). Cells were stained with FxCycle™ PI/RNase Staining Solution (Thermo Fisher Scientific, Waltham, MA, USA). While the included RNase digests the RNA of the fixed cells, the DNA content is stained with propidium iodide. Emission signals are proportional to DNA mass. Signal peaks for G0/G1 phase (one set of paired chromosomes per cell) and G2/M phase peak (two sets of paired chromosomes per cell, prior to cell division) are separated by S phase signals (DNA synthesis with variable amount of DNA). An early time point (12 h) was chosen to minimize the influence of mitotic slippage and its formation of cells with irregular DNA content, which may interfere with the results. 

PC-3 and 0846 cells were seeded in 6-well plates (120 × 10^3^ cells per well) and incubated in 3 mL culture medium overnight. The culture medium was replaced by new medium including 5 µM FX-9 or DMSO as control. After 12 h, the cells were washed with PBS, harvested, and pelleted. The cells were resuspended with 100 µL PBS and afterwards fixed by slowly adding 1 mL 70% ethanol (−20 °C) and an incubation of 20 min at 4 °C. Cells were washed with PBS again and pelleted. Finally, cells were resuspended in 500 µL FxCycle™ PI/RNase Staining Solution. Before measurement, the samples were incubated at room temperature for 30 min in the dark. The results were analyzed with FlowJo Version 7.6.5 (FlowJo, Ashland, OR, USA). The experiment was carried out in biological triplicates.

### 4.9. Statistical Analysis

Within each experiment, results were described using mean ± standard deviation (SD). Significant differences between treatment and control as well as the malignant and benign cells were calculated by Dunnett’s Multiple Comparison Test or Student’s *t*-test. Differences were considered statistically significant for *p* < 0.05. All tests were performed with SAS software 7.1 (SAS Institute Inc., Cary, NC, USA).

## Figures and Tables

**Table 1:** PC-3 and 0846 cells were exposed to 5 µM FX-9 for 12 h. Analysis of cell cycle was performed using propidium iodide staining and RNA digestion of fixed cells with subsequent flow cytometry analysis. The table shows the mean of three independent experiments. Significance of a treatment effect compared to the respective DMSO-treated negative control value was determined using Student’s *t*-test. *: *p* < 0.05.

Cell Line	PC-3		0846	
Application	Control	12 h of 5 µM FX-9	Control	12 h of 5 µM FX-9
G0/G1 phase [%]	36.2	31.5 *	39.5	32.3 *
S phase [%]	24.4	19.5 *	21.7	17.9 *
G2/M phase [%]	39.4	49.0 *	38.8	49.8 *

**Table 2:** Comparison between malignant cell lines and benign cells after 72 h of FX-9 application. The table shows the mean values of the performed assays. Missing CT1258 values: the cell line was exposed to 2.5 µM for these assays. The four PCa cell lines were compared to each benign cells by Dunnett’s Multiple Comparison Test. *: Significant difference compared to both benign cell types; *p* < 0.05. ^1^: Significant difference to hTF-8 only; *p* < 0.05.

Cellular Analysis	Malignant Cell Lines				Benign Cells	
	PC-3	LNCaP	CT1258	0846	hTF-8	1801
Cell viability (5 µM FX-9) [%]	38.7 *	31.8 *	29.6 *	58.4	77.8	78.8
Cell viability (7.5 µM FX-9) [%]	35.2 *	33.9 *	30.7 *	42.5 *	74.5	69.8
Cell viability (10 µM FX-9) [%]	35.6 ^1^	27.4 *	27.8 *	37.0 ^1^	66.9	60.1
Total cell count (5 µM FX-9) [%]	14.1 *	13.4 *	−	7.6 *	95.2	74.4
Vital cells (5 µM FX-9) [%]	33.3 *	13.0 *	−	23.2 *	67.5	75.9
Apoptotic cells (5 µM FX-9) [%]	59.1 *	80.8 *	−	71.0 *	23.0	13.8

## Supplementary Materials

The supplementary materials are available online at https://www.mdpi.com/1422-0067/20/22/5567/s1.

## Abbreviations

PCa	prostate cancer
ALL	acute lymphoblastic leukemia
DMSO	dimethyl sulfoxide
SD	standard deviation
